# Deriving the PedsUtil health state classification system to measure health utilities for pediatric populations based on the PedsQL: a confirmatory factor analysis

**DOI:** 10.1186/s12955-024-02300-8

**Published:** 2024-10-08

**Authors:** Ellen Kim DeLuca, Kim Dalziel, Eve Wittenberg, Angela M. Rose, Lisa A. Prosser

**Affiliations:** 1 Department of Population Medicine, Harvard Pilgrim Health Care Institute, Boston, MA USA; 2 Department of Health Management and Policy, Michigan School of Public Health, University of Michigan, Ann Arbor, MI USA; 3 Centre for Health Policy, Melbourne School of Population and Global Health, University of Melbourne, Parkville, VIC Australia; 4 Center for Health Decision Science, Harvard T.H. Chan School of Public Health, Harvard University, Boston, MA USA; 5 Susan B. Meister Child Health Evaluation & Research Center, Department of Pediatrics, Michigan Medicine, University of Michigan, Ann Arbor, MI USA

**Keywords:** Health-related quality of life, PedsQL, Pediatrics, Factor analysis

## Abstract

**Background:**

An important methodological challenge in conducting pediatric economic evaluations is estimating the preference-based health-related quality of life (HRQoL) of children. Current methods are highly variable and there is no single instrument available to value HRQoL consistently across multiple pediatric age groups. The Pediatric Quality of Life Inventory (PedsQL) is a non-preference-based generic HRQoL instrument validated for children 2–18 years, but it cannot be directly used in economic evaluations. The aim of this study was to establish the core dimension structure of the PedsUtil health state classification system using confirmatory factor analysis, which is the first step of deriving a preference-based measure of HRQoL based on the PedsQL.

**Methods:**

Four competing dimension structures of the PedsUtil health state classification system were developed based on published literature and expert opinion. Using data from the Longitudinal Study of Australian Children (LSAC) (*n* = 45,207), the 4 dimension structures were evaluated using the robust weighted least squares estimation method. The analyses were stratified by 2-year age intervals (from 2 to 17 years) to reflect the study design of the LSAC, as well as special healthcare needs status of the child. Model fit was evaluated by examining standardized factor loadings and various fit indices including the comparative fit index (CFI), Tucker-Lewis Index (TLI), and the root mean square error of approximation (RMSEA). Modification indices and residual correlations were examined to re-specify the models to improve model fit when necessary.

**Results:**

The findings supported a 7-dimension structure (i.e., Physical Functioning, Pain, Fatigue, Emotional Functioning, Social Functioning, School Functioning, and School Absence) of the PedsUtil health state classification system. The 7-dimension model exhibited adequate fit across subgroups with CFI values that ranged from 0.929 to 0.954, TLI values from 0.916 to 0.946, and RMSEA values from 0.058 to 0.102.

**Conclusions:**

This study established the core dimension structure of the PedsUtil health state classification system using confirmatory factor analysis. The 7-dimension structure was found to be applicable across diverse pediatric populations. Research is currently ongoing to select the most representative item within each dimension of the PedsUtil health state classification system and valuation surveys will be fielded to estimate the PedsUtil scoring system.

**Supplementary Information:**

The online version contains supplementary material available at 10.1186/s12955-024-02300-8.

## Background

Preference-based measures of health-related quality of life (HRQoL) can be utilized in health economic evaluations to calculate quality-adjusted life years (QALYs), the standard metric for valuing health in cost-effectiveness analyses. However, methodological developments to value child HRQoL have lagged behind comparable research for valuing adult HRQoL. A critical limitation is the paucity of child-specific preference-based measures of HRQoL [1–5]. Currently, there are very few valid and reliable preference-based measures of HRQoL that can be applied across multiple pediatric age groups. Most available measures have either been developed for adult populations and applied to children without adjustment [6–8] or apply only to limited age groups [9, 10]. Therefore, there is a need for a single preference-based measure of HRQoL that can be applied across a wide range of pediatric age groups in order to achieve greater consistency in valuing child health.

The Pediatric Quality of Life Inventory (PedsQL) is a generic HRQoL instrument widely used in pediatric clinical trials and is validated for children 2–18 years [11, 12]. However, the PedsQL is not a preference-based measure, thus it cannot be directly used to calculate QALYs in cost-effectiveness analyses. The PedsQL provides a simple summative score that describes HRQoL, whereas QALYs are derived by combining the time spent in a health state with the corresponding health utility or preference weight for that health state [13]. Given that the PedsQL offers summative scores rather than preference-based values (health utilities), it cannot be used to calculate QALYs. One solution to expand its use into cost-effectiveness analyses is to develop a health utility scoring system for the PedsQL: the PedsUtil scoring system.

Constructing the PedsUtil scoring system is a multi-step process that first requires deriving a health state classification system based on the PedsQL that is amenable to preference elicitation methods. Specifically, the PedsQL includes more items than is manageable to value in the preference elicitation exercise required to construct the PedsUtil scoring system. With 23 items, each ranging 5 severity levels from “Never” to “Almost always” [11, 12], the PedsQL in its entirety would generate 5^23^ health states, which are feasibly too many to value. Therefore, it is necessary to reduce the length of the original instrument to a core set of dimensions and items to form the PedsUtil health state classification system. This approach to constructing a health state classification system from an existing non-preference-based measure has been previously used to derive preference-based measures of adult generic HRQoL and condition-specific HRQoL [14, 15], such as the development of the SF-6D from the SF-36 [7, 16] and the QLU-C10D from the QLQ-C30 [17].

The objective of this study was to conduct the first step in deriving the PedsUtil health state classification system by establishing its core dimension structure using confirmatory factor analysis. One requirement of a multidimensional health state classification system is that the dimensions are structurally independent (i.e., dimensions are orthogonal) to avoid nonsensical health states [14]. While various techniques, such as principal component analysis, exploratory factor analysis, and cluster analysis, can be used to help identify structurally independent dimensions, confirmatory factor analysis allows for testing of a specific dimensional structure (i.e., the conceptual model). The advantage of this approach is that theoretically or clinically driven decisions can be integrated into the general method of item assessment at the outset, rather than as an afterthought [18]. Given that the PedsQL is a well-validated instrument with a previously established dimensional structure, confirmatory factor analysis was deemed most appropriate for identifying the dimensionality of a health state classification system based on the PedsQL.

## Methods

### The PedsQL

The PedsQL 4.0 Generic Core Scales is a modular instrument that measures generic HRQoL in children and adolescents from ages 2 to 18 years [11, 12]. It was developed through focus groups and cognitive interviews, and consists of child self-report and parent proxy-report versions. The child self-report version includes age groups 5–7 years, 8–12 years and 13–18 years. The parent proxy-report version includes age groups 2–4 years, 5–7 years, 8–12 years, and 13–18 years. The items in the different versions are very similar and differ only in developmentally appropriate vocabulary and first or third person tense. A 5-point response scale (0 = Never; 1 = Almost never; 2 = Sometimes; 3 = Often; 4 = Almost always) is used for child self-report ages 8 years and older, and for all age groups with the parent proxy-report. For child self-report ages 5 to 7 years, a 3-point response scale (0 = Not at all; 2 = Sometimes; 4 = A lot) is used to increase comprehension. The PedsQL Infant Scales, which is validated to evaluate HRQoL for infants 1–24 months [19], was not included in this analysis as it consists of different items and dimensions than the PedsQL Generic Core Scales.

Overall, there are 21 to 23 multi-level items that fall under 4 different dimensions or subscales: (1) Physical Functioning; (2) Emotional Functioning; (3) Social Functioning; and (4) School Functioning (Table 1). The items are reverse-scored and linearly transformed to a 0 to 100 scale (0 = 100; 1 = 75; 2 = 50; 3 = 25; 4 = 0), where higher scores indicate better HRQoL. The total HRQoL score is calculated as the sum of the completed item scores divided by the number of items answered [11].

**Table 1:** Summary of PedsQL

Dimensions	Number of Items			
	2–4 years	5–7 years	8–12 years	13–18 years
Physical Functioning	8	8	8	8
Emotional Functioning	5	5	5	5
Social Functioning	5	5	5	5
School Functioning	3	5	5	5
**Total**	21^a^	23	23	23

^a^PedsQL School Functioning contains 2 fewer items for children 2–4 years to reflect developmentally appropriate items

### Data source

This study analyzed data from the Longitudinal Study of Australian Children (LSAC), which is a study that follows a population-representative sample of approximately 10,000 children and their families [20]. The LSAC delivers a comprehensive national dataset on children as they age and is one of the very few large-scale nationally representative surveys of children in the world. The LSAC sampling design is detailed elsewhere [21]. The LSAC was approved by the Australian Institute of Family Studies Ethics Committee, and families provided written informed consent to participate [22].

This study used data from the first 7 waves (2003-04 to 2015-16) of the LSAC (*n* = 45,207 observations from 9,262 children). This dataset contains fully completed responses to the parent proxy-report version of the PedsQL at each wave of data collection for the same children at different ages from 2 to 17 years. The only exception was for children 2–3 years old, in which responses to only 19 of the 21 PedsQL items were available; the 2 items on school absence were not collected as part of the LSAC. Consequently, all analyses conducted in this study for children aged 2–3 years only utilized the 19 PedsQL items that were administered. For all other age groups, responses to the full PedsQL were available. Special healthcare needs status of LSAC participants were also categorized in this study as children with special healthcare needs or typically functioning children. A child was considered to have special healthcare needs if a parent reported that their child had “a condition which has lasted or is expected to last for at least 12 months, which causes the child to use medicine prescribed by a doctor, other than vitamins, or use more medical care, mental health or educational services” [23]. Data from the last available wave for each child was used to determine special healthcare needs status since younger children are less likely to be identified as having special healthcare needs.

### Statistical analysis – confirmatory factor analysis

Four main competing conceptual models of the PedsUtil health state classification system were developed a priori, drawing on published literature and expert opinion (Table 2). The first conceptual model, Model A, was the original 4-dimension structure of the PedsQL: Physical Functioning, Emotional Functioning, Social Functioning, and School Functioning. The second, Model B, was comprised of 5 dimensions: Physical Functioning, Emotional Functioning, Social Functioning, School Functioning, and School Absence. Model B was specified based on previously published literature that found model fit to be better when School Functioning items were split into 2 separate dimensions [11, 12, 24]; specifically, the 5-item School Functioning dimension was split into one dimensions with 3 items measuring school cognitive functioning and a second dimension with 2 items measuring school absence as related to illness. The third conceptual model, Model C, included 6 dimensions, 2 of which were single-item dimensions: Physical Functioning, Pain, Fatigue, Emotional Functioning, Social Functioning, and School Functioning. Model C was constructed based on expert opinion and by referencing other available preference-based HRQoL instruments for children [8–10, 25, 26] that suggested that the items measuring Pain and Fatigue (originally in the Physical Functioning dimension) may be clinically important to distinguish as independent dimensions. Lastly, Model D was composed of 7 dimensions, and was formed by combining Models B and C: Physical Functioning, Pain, Fatigue, Emotional Functioning, Social Functioning, School Functioning, and School Absence.

**Table 2:** Summary of dimension structures for conceptual models A-D

Item^a^ Problems with…	Model A (4 Dimensions)	Model B (5 Dimensions)	Model C (6 Dimensions)	Model D (7 Dimensions)
Phys 1. Walking	Physical	Physical	Physical	Physical
Phys 2. Running	Physical	Physical	Physical	Physical
Phys 3. Participating in exercise	Physical	Physical	Physical	Physical
Phys 4. Lifting something heavy	Physical	Physical	Physical	Physical
Phys 5. Taking a bath or shower	Physical	Physical	Physical	Physical
Phys 6. Doing chores	Physical	Physical	Physical	Physical
Phys 7. Having hurts or aches	Physical	Physical	Pain^b^	Pain^b^
Phys 8. Low energy level	Physical	Physical	Fatigue^b^	Fatigue^b^
Emot 1. Feeling afraid or scared	Emotional	Emotional	Emotional	Emotional
Emot 2. Feeling sad or blue	Emotional	Emotional	Emotional	Emotional
Emot 3. Feeling angry	Emotional	Emotional	Emotional	Emotional
Emot 4. Trouble sleeping	Emotional	Emotional	Emotional	Emotional
Emot 5. Worrying	Emotional	Emotional	Emotional	Emotional
Soc 1. Getting along with others	Social	Social	Social	Social
Soc 2. Others not wanting to be friends	Social	Social	Social	Social
Soc 3. Getting teased	Social	Social	Social	Social
Soc 4. Unable to do things others can do	Social	Social	Social	Social
Soc 5. Keeping up with other children	Social	Social	Social	Social
School 1. Paying attention in class	School	School	School	School
School 2. Forgetting things	School	School	School	School
School 3. Keeping up with schoolwork	School	School	School	School
SchAbs 1. Missing school because sick	School	School Absence	School	School Absence
SchAbs 2. Missing school to go to doctor	School	School Absence	School	School Absence

*Abbreviations* *Phys* Physical Functioning; *Emot* Emotional Functioning; *Soc* Social Functioning; *School* School Functioning; *SchAbs* School Absence

^a^Item descriptions are summarized in the table (exact wording of items not displayed)

^b^Single-item dimensions (i.e., Pain and Fatigue) are included in the conceptual models but could not be empirically tested, thus are omitted from the measurement models

The 4 conceptual models were evaluated using the mean- and variance-adjusted weighted least squares estimation method (estimator = WLSMV), which is recommended for modeling ordinal data [27]. However, the single-item dimensions of Pain and Fatigue, which were included a priori in conceptual Models C and D due to their clinical importance, could not be empirically tested. This is because confirmatory factor analysis requires at least 2 items in a dimension to empirically estimate the measurement model. Therefore, items for Pain and Fatigue were omitted from the empirical measurement model despite their inclusion in the conceptual models. Because the PedsUtil health state classification should ideally be applicable across diverse pediatric populations, analyses were stratified by age group and child special healthcare needs status in order to identify a common dimension structure across all subgroups. Age groups were stratified by 2-year age intervals (from 2 to 17 years) to reflect the study design of the LSAC. Special healthcare needs status was defined as children with special healthcare needs or typically functioning children.

Model fit was evaluated in this study by examining standardized factor loadings and various fit indices including the comparative fit index (CFI) [28], Tucker-Lewis Index (TLI) [29], and the root mean square error of approximation (RMSEA) [30]. Previously established guidelines suggest adequate fitting models have CFI and TLI values ≥ 0.90 and RMSEA values ≤ 0.08 [31]. Modification indices (output = modindices) and residual correlations (output = residual) were examined to re-specify the models to improve model fit when necessary. Models were modified and re-fit until a clinically coherent model was achieved across all subgroups that also adequately fit the data. All analyses were conducted in Mplus v8 [27]. This study was determined to be exempt by the University of Michigan Institutional Review Board (IRBMED # HUM00182088).

## Results

### Sample characteristics

Table 3 presents characteristics of the LSAC participants by child special healthcare needs status. As expected, the individual PedsQL scale scores and total score were lower for children with special healthcare needs than for typically functioning children. Children with special healthcare needs had an average total score of 74.8 and typically functioning children had an average total score of 81.0. In addition, most of the parent respondents were female (96%) and had earned at least a high school degree (70%).

**Table 3:** Summary of LSAC participants by child special healthcare needs status

Characteristic	Children with Special Healthcare Needs (*n* = 8,793)	Typically Functioning Children^a^ (*n* = 36,414)
**Child**		
Age, y		
Mean (SD)	9.1 (4.1)	8.8 (4.0)
Age Distribution [*n* (%)]		
2–3 years	519 (5.9)	2,615 (7.2)
4–5 years	1,330 (15.1)	5,862 (16.1)
6–7 years	1,343 (15.3)	5,765 (15.8)
8–9 years	1,380 (15.7)	5,938 (16.3)
10–11 years	1,450 (16.5)	5,933 (16.3)
12–13 years	1,315 (15.0)	5,434 (14.9)
14–15 years	775 (8.8)	2,588 (7.1)
16–17 years	681 (7.7)	2,279 (6.3)
Sex [*n* (%)]		
Male	4,642 (52.8)	18,482 (50.8)
Female	4,151 (47.2)	17,932 (49.2)
PedsQL Scale Scores [mean (SD)]		
Physical Functioning	78.9 (17.2)	83.9 (14.7)
Emotional Functioning	68.8 (18.0)	75.8 (15.3)
Social Functioning	76.7 (19.9)	83.6 (15.7)
School Functioning	73.0 (20.0)	79.8 (16.8)
Total Score	74.8 (14.8)	81.0 (11.9)
**Parent** ^b^		
Age, y		
Mean (SD)	40.3 (6.8)	39.9 (6.5)
Sex [*n* (%)]		
Male (%)	344 (3.9)	1,366 (3.8)
Female (%)	8,449 (96.1)	35,048 (96.3)
Education [*n* (%)]		
Less than high school	117 (1.3)	387 (1.1)
Some high school	2,649 (30.2)	10,510 (28.9)
High school graduate	2,807 (31.9)	12,431 (34.2)
College degree	1,704 (19.4)	6,981 (19.2)
Graduate degree	1,510 (17.2)	6,065 (16.7)

*Abbreviations* *LSAC* Longitudinal Study of Australian Children; *y* years; *SD* standard deviation

^a^Typically functioning children excluded children with special healthcare needs

^b^Parent that answered the PedsQL about their child

### Confirmatory factor analysis

Table 4 reports the confirmatory factor analysis fit indices for Models A-D across all age and child special healthcare needs status subgroups. Models A-C generally did not meet the cutoffs for the various model fit indices and model fit was worse for children with special healthcare needs than for typically functioning children. Model A had CFI values that ranged from 0.879 to 0.918, TLI values from 0.863 to 0.912, and RMSEA values from 0.071 to 0.118; Model B had CFI values from 0.906 to 0.931, TLI values from 0.892 to 0.920, and RMSEA values from 0.067 to 0.110; and Model C had CFI values from 0.898 to 0.945, TLI values from 0.881 to 0.937, and RMSEA values from 0.087 to 0.111. On the other hand, Model D exhibited adequate model fit for most subgroups with CFI values that ranged from 0.929 to 0.954, TLI values from 0.916 to 0.946, and RMSEA values from 0.058 to 0.102. Additionally, despite the acceptable fit for Model D across most subgroups, model fit was slightly better for typically functioning children than for children with special healthcare needs. Table a1 in the Supplement also presents the confirmatory factor analysis fit indices when children with special healthcare needs and typically functioning children were combined across age groups, and this analysis similarly found Model D to be the best fitting model.

Table 5 displays the standardized factor loadings for Model D for all subgroups. All items had salient factor loadings > 0.4 and *p*-values < 0.001, though 14 out of the 21 items had factor loadings > 0.7 across all subgroups. In addition, certain items (Phys 4–6, Emot 1–4, Soc 1–5, and School 2) exhibited a general trend for higher factor loadings as children increased in age (Table a2).

Though model fit was generally adequate for Model D, the RMSEAs were > 0.08 for all age groups 6 years and older for children with special healthcare needs and age groups 10–11 years and 14–15 years for typically functioning children. When modification indices and residual correlations were examined for age groups 6 years and older, items Soc 1 (“getting along with others”), Soc 4 (“unable to do things others can do”), and Soc 5 (“keeping up with other children”) appeared to cross-load onto the Physical Functioning dimension. Therefore, Model D was re-specified with these cross-loadings and the re-specified model fit indices are shown in Table 6. Similar to the original model, fit indices for the re-specified model indicated slightly better model fit for typically functioning children (CFI ≥ 0.96, TLI ≥ 0.952, and RMSEA ≤ 0.071) than for children with special healthcare needs (CFI ≥ 0.954, TLI ≥ 0.945, and RMSEA ≤ 0.076) across age groups. Nevertheless, model fit improved across all subgroups, resulting in model fit indices that were considered acceptable (i.e., cutoff criteria of CFI and TLI ≥ 0.90 and RMSEA ≤ 0.08).

Although overall model fit improved when items Soc 1, Soc 4, and Soc 5 were cross-loaded onto the Physical Functioning dimension, these items need to be allocated to a single dimension in order to create the PedsUtil health state classification system. Therefore, these items were retained in the Social Functioning dimension as originally hypothesized, and Model D was determined to be the core dimension structure of the PedsUtil health state classification system.

**Table 4:** Confirmatory factor analysis fit indices for models A-D across subgroups

Fit Indices	Children with Special Healthcare Needs								Typically Functioning Children							
	2/3 years^a^	4/5 years	6/7 years	8/9 years	10/11 years	12/13 years	14/15 years	16/17 years	2/3 years^a^	4/5 years	6/7 years	8/9 years	10/11 years	12/13 years	14/15 years	16/17 years
**Model A**																
RMSEA	0.087	0.107	0.113	0.112	0.117	0.112	0.118	0.109	0.071	0.092	0.101	0.098	0.107	0.094	0.109	0.103
RMSEA 90% CI	0.080–0.093	0.104–0.111	0.110–0.116	0.109–0.115	0.114–0.120	0.109–0.115	0.114–0.122	0.105–0.114	0.068–0.074	0.091–0.094	0.099–0.102	0.097-0.100	0.106–0.108	0.092–0.095	0.107–0.111	0.101–0.105
CFI	0.907	0.881	0.885	0.879	0.892	0.898	0.909	0.908	0.918	0.887	0.902	0.889	0.896	0.906	0.922	0.902
TLI	0.892	0.863	0.870	0.864	0.878	0.885	0.897	0.896	0.905	0.870	0.890	0.875	0.882	0.894	0.912	0.890
**Model B**																
RMSEA	–	0.082	0.101	0.098	0.110	0.104	0.109	0.102	–	0.067	0.089	0.088	0.100	0.085	0.104	0.098
RMSEA 90% CI	–	0.079–0.086	0.098–0.104	0.095–0.101	0.107–0.113	0.101–0.107	0.105–0.113	0.097–0.106	–	0.066–0.069	0.088–0.091	0.087–0.090	0.098–0.101	0.083–0.087	0.102–0.106	0.095-0.100
CFI	–	0.912	0.910	0.908	0.906	0.914	0.923	0.921	–	0.929	0.925	0.913	0.911	0.924	0.931	0.914
TLI	–	0.899	0.897	0.895	0.892	0.901	0.912	0.909	–	0.917	0.914	0.900	0.898	0.913	0.920	0.901
**Model C**																
RMSEA	–	0.109	0.110	0.111	0.111	0.104	0.109	0.099	–	0.093	0.096	0.094	0.100	0.087	0.100	0.088
RMSEA 90% CI	–	0.105–0.113	0.107–0.113	0.108–0.114	0.108–0.115	0.100-0.107	0.104–0.113	0.091-0.100	–	0.092–0.095	0.094–0.096	0.093–0.096	0.098–0.102	0.085–0.089	0.098–0.102	0.085–0.090
CFI	–	0.898	0.909	0.898	0.917	0.925	0.934	0.930	–	0.901	0.925	0.912	0.923	0.929	0.945	0.937
TLI	–	0.881	0.896	0.882	0.904	0.913	0.925	0.920	–	0.885	0.913	0.899	0.912	0.919	0.937	0.928
**Model D**																
RMSEA	0.077	0.072	0.093	0.094	0.102	0.093	0.096	0.089	0.061	0.058	0.079	0.080	0.089	0.075	0.092	0.078
RMSEA 90% CI	0.070–0.085	0.068–0.076	0.089–0.096	0.090–0.097	0.098–0.105	0.090–0.097	0.092–0.101	0.084–0.094	0.057–0.064	0.057–0.060	0.077–0.080	0.078–0.081	0.087–0.090	0.073–0.076	0.090–0.095	0.076–0.081
CFI	0.937	0.942	0.937	0.929	0.932	0.941	0.950	0.948	0.947	0.953	0.950	0.939	0.941	0.949	0.954	0.951
TLI	0.925	0.932	0.926	0.916	0.920	0.930	0.941	0.939	0.937	0.945	0.942	0.928	0.930	0.940	0.946	0.943

*Abbreviations* *RMSEA* root mean square error of approximation; *CFI* comparative fit index; *TLI* Tucker-Lewis index; *CI* confidence interval

^a^ For children aged 2–3 years, the measurement models for Models A and B and for Models C and D were the same because only 1 School Functioning item was included in the LSAC dataset

**Table 5:** Factor loadings for model D across subgroups

Item	Children with Special Healthcare Needs								Typically Functioning Children							
	2/3 years	4/5 years	6/7 years	8/9 years	10/11 years	12/13 years	14/15 years	16/17 years	2/3 years	4/5 years	6/7 years	8/9 years	10/11 years	12/13 years	14/15 years	16/17 years
Phys 1	0.867	0.872	0.888	0.904	0.894	0.882	0.900	0.880	0.907	0.910	0.911	0.877	0.933	0.902	0.932	0.902
Phys 2	0.921	0.947	0.950	0.917	0.913	0.914	0.902	0.910	0.864	0.944	0.972	0.922	0.932	0.903	0.917	0.872
Phys 3	0.824	0.843	0.925	0.896	0.933	0.929	0.943	0.918	0.753	0.807	0.934	0.920	0.943	0.923	0.946	0.902
Phys 4	0.618	0.625	0.761	0.749	0.801	0.809	0.848	0.807	0.566	0.590	0.703	0.686	0.753	0.783	0.847	0.858
Phys 5	0.631	0.637	0.716	0.727	0.825	0.794	0.849	0.773	0.573	0.686	0.766	0.745	0.890	0.837	0.957	0.915
Phys 6	0.478	0.416	0.604	0.621	0.613	0.654	0.759	0.602	0.567	0.492	0.633	0.661	0.680	0.646	0.774	0.631
Pain^a^	–	–	–	–	–	–	–	–	–	–	–	–	–	–	–	–
Fatigue^a^	–	–	–	–	–	–	–	–	–	–	–	–	–	–	–	–
Emot 1	0.693	0.701	0.751	0.786	0.795	0.804	0.857	0.858	0.655	0.718	0.730	0.743	0.785	0.771	0.811	0.814
Emot 2	0.712	0.733	0.755	0.789	0.817	0.847	0.872	0.833	0.713	0.732	0.756	0.788	0.792	0.826	0.847	0.848
Emot 3	0.621	0.593	0.688	0.675	0.698	0.749	0.789	0.705	0.642	0.621	0.638	0.667	0.688	0.692	0.768	0.728
Emot 4	0.481	0.535	0.622	0.617	0.641	0.640	0.727	0.691	0.482	0.492	0.566	0.605	0.591	0.617	0.676	0.676
Emot 5	0.800	0.791	0.708	0.747	0.788	0.789	0.818	0.801	0.785	0.775	0.704	0.740	0.758	0.790	0.801	0.811
Soc 1	0.764	0.748	0.749	0.751	0.780	0.803	0.785	0.750	0.708	0.725	0.773	0.765	0.787	0.777	0.810	0.818
Soc 2	0.730	0.782	0.779	0.832	0.878	0.860	0.865	0.850	0.787	0.771	0.736	0.814	0.808	0.833	0.835	0.844
Soc 3	0.744	0.706	0.712	0.780	0.804	0.814	0.840	0.794	0.723	0.733	0.719	0.789	0.783	0.789	0.839	0.808
Soc 4	0.793	0.802	0.802	0.821	0.816	0.804	0.847	0.859	0.764	0.735	0.695	0.749	0.714	0.781	0.801	0.784
Soc 5	0.838	0.821	0.872	0.859	0.855	0.835	0.870	0.902	0.756	0.767	0.892	0.823	0.877	0.844	0.854	0.863
School 1	N/A^b^	N/A^b^	0.857	0.857	0.867	0.892	0.883	0.903	N/A^b^	N/A^b^	0.838	0.862	0.848	0.877	0.918	0.874
School 2	N/A^b^	N/A^b^	0.716	0.752	0.729	0.764	0.766	0.755	N/A^b^	N/A^b^	0.646	0.702	0.660	0.765	0.744	0.751
School 3	–^c^	–^c^	0.961	0.894	0.942	0.867	0.889	0.864	–^c^	–^c^	0.957	0.904	0.930	0.874	0.891	0.854
SchAbs 1	N/A^d^	0.865	0.795	0.792	0.784	0.752	0.876	0.877	N/A^d^	0.873	0.785	0.811	0.823	0.812	0.827	0.874
SchAbs 2	N/A^d^	0.898	0.915	0.921	0.883	0.917	0.867	0.823	N/A^d^	0.855	0.917	0.863	0.881	0.838	0.830	0.794

*Abbreviations* *Phys* Physical Functioning; *Emot* Emotional Functioning; *Soc* Social Functioning; *School* School Functioning; *SchAbs* School Absence; *N/A* not applicable

^a^Pain and Fatigue are single item dimensions so could not be empirically tested using confirmatory factor analysis

^b^School 1 and School 2 are not included in the parent proxy-report version of the PedsQL for young children

^c^School 3 was a single item dimension for children aged 2–5 years since School 1 and School 2 are not included in the PedsQL for those age groups, thus the School Functioning dimension could not be empirically tested using confirmatory factor analysis for age groups 2–3 years and 4–5 years

^d^SchAbs 1 and SchAbs 2 were not administered for children aged 2–3 years in the LSAC

**Table 6:** Fit indices for re-specified model D^a^

Fit Indices	Children with Special Healthcare Needs						Typically Functioning Children					
	6/7 years	8/9 years	10/11 years	12/13 years	14/15 years	16/17 years	6/7 years	8/9 years	10/11 years	12/13 years	14/15 years	16/17 years
RMSEA	0.072	0.076	0.074	0.076	0.074	0.074	0.061	0.065	0.062	0.060	0.071	0.067
RMSEA 90% CI	0.068–0.075	0.073–0.080	0.071–0.077	0.072–0.079	0.069–0.079	0.069–0.079	0.060–0.063	0.063–0.067	0.061–0.064	0.058–0.061	0.068–0.073	0.064–0.070
CFI	0.963	0.954	0.965	0.961	0.971	0.964	0.970	0.960	0.971	0.968	0.973	0.965
TLI	0.955	0.945	0.958	0.954	0.965	0.957	0.965	0.952	0.966	0.962	0.968	0.958

*Abbreviations* *RMSEA* root mean square error of approximation; *CFI* comparative fit index; *TLI* Tucker-Lewis index; *CI* confidence interval

^a^Model D re-specified by cross-loading items Soc 1 (“getting along with others”), Soc 4 (“unable to do things others can do”), and Soc 5 (“keeping up with other children”) onto the Physical Functioning dimension

## Discussion

The first step in deriving a preference-based HRQoL measure from a non-preference-based instrument is to construct a health state classification system that is amenable to preference elicitation methods. This study conducted confirmatory factor analysis to identify the core dimension structure of the PedsUtil health state classification system based on the PedsQL. Among the four conceptual models hypothesized, the 7-dimension structure of the PedsUtil health state classification system (i.e., Physical Functioning, Pain, Fatigue, Emotional Functioning, Social Functioning, School Functioning, and School Absence) exhibited the best model fit. The subgroup analyses also suggest that this 7-dimension structure may be applicable across diverse pediatric populations, including children with special healthcare needs and typically functioning children, as well as children aged 2–17 years.

Previous studies have examined the dimension structure of the PedsQL using confirmatory factor analysis across various pediatric populations. Consistent with the findings of this study, these studies demonstrated that splitting the 5 items in the original School Functioning dimension into 2 separate dimensions – School Functioning and School Absence – generally exhibited better model fit [24, 32–36). This led to identifying the 5-dimension structure of the PedsQL (i.e., Physical Functioning, Emotional Functioning, Social Functioning, School Functioning, and School Absence) as the most appropriate. However, none of the previous studies evaluated the dimension structure of the PedsQL with Pain and Fatigue as single-item dimensions as done in this analysis.

Though the 7-dimension structure was the best fitting model across all subgroups in this study, some trends in the factor loadings and model fit indices were observed. For example, some items exhibited higher factor loadings for older children. This positive trend suggests that the construct validity of some items may improve for older children, given that they may have increased opportunities to experience or express some of the behaviors described by the PedsQL items. Additionally, model fit was slightly better for typically functioning children than for children with special healthcare needs. This may be because there is more homogeneity in the responses of typically functioning children, leading to a more consistent pattern that aligns with the hypothesized 7-dimension model structure. Previous studies have also examined factorial invariance for the PedsQL across pediatric subpopulations. These studies similarly found that PedsQL items are comparable across age [32] and child health status subgroups [33], as well as across various race [34], gender [35], and socioeconomic status subgroups [36].

There are some potential limitations to this study. First, this study was conducted using parent-proxy responses to the PedsQL from an Australian population, as the LSAC is one of the most extensive pediatric datasets available with PedsQL responses. Future research should validate dimensionality of the health state classification system using data from other populations, as well as the child self-report version of the PedsQL for age groups 5–7 years, 8–12 years, and 13–18 years. Second, this study did not empirically estimate any single-item dimensions (i.e., Pain and Fatigue). While it may be possible to model these single-item dimensions through higher-order models, this approach was not taken due to the potential issues regarding the causal structure among dimensions and the limited relevance of higher-order models in identifying the core dimension structure of the PedsUtil health state classification system. Subsequent research will incorporate alternative methods, including psychometric analyses and expert and parent opinion, to further validate the clinical importance and inclusion of Pain and Fatigue as single-item dimensions in the health state classification system. Lastly, this study did not test for metric and scalar invariance across subgroups, which are stricter conditions of measurement invariance. Given that the objective was to identify a conceptually sound dimension structure where each common dimension is associated with identical sets of items across various pediatric subgroups, measurement models for each subgroup were estimated separately to establish configural invariance.

## Conclusions

This study established the core dimension structure of the PedsUtil health state classification system using confirmatory factor analysis, which is the first key step to developing the PedsUtil scoring system. The 7-dimension structure of the PedsUtil health state classification system (Model D: Physical Functioning, Pain, Fatigue, Emotional Functioning, Social Functioning, School Functioning, and School Absence) demonstrated the best model fit across diverse pediatric subgroups, including children with special healthcare needs and typically functioning children, as well as children aged 2–17 years. Following research includes selecting the most representative item within each dimension of the PedsUtil health state classification system [37] and fielding valuation surveys to estimate the PedsUtil scoring system. The development of the PedsUtil health state classification system and its resulting value set will ensure that children’s experiences with disease and treatment are consistently and accurately represented in healthcare value assessments, supporting evidence-based decisions for healthcare priority setting.

## Electronic supplementary material

Below is the link to the electronic supplementary material.

Supplementary Material 1

## Data Availability

The data that support the findings of this study are available from Australian Data Archive (ADA), but restrictions apply to the availability of these data, which were used under license for the current study and so are not publicly available. The data are, however, available from the authors upon reasonable request and with permission of Australian Data Archive.

## Abbreviations

HRQoL: Health-related quality of life
QALY: Quality-adjusted life year
PedsQL: Pediatric Quality of Life Inventory
LSAC: Longitudinal Study of Australian Children
Phys: Physical Functioning
Emot: Emotional Functioning
Soc: Social Functioning
School: School Functioning
SchAbs: School Absence
CFI: Comparative fit index
TLI: Tucker-Lewis Index
RMSEA: Root mean square error of approximation
