# Supplementary material for: Deriving the PedsUtil health state classification system to measure health utilities for pediatric populations based on the PedsQL: a confirmatory factor analysis
Source: Health Qual Life Outcomes. 2024 Oct 8;22:85. doi: 10.1186/s12955-024-02300-8 (PMC11463099; doi:10.1186/s12955-024-02300-8)
Supplement: Supplementary file 1 — Supplementary Material 1 [file 12955_2024_2300_MOESM1_ESM.docx]

**Table a1. Confirmatory Factor Analysis Fit Indices for Models A-D Across Age Groups for All Children**

| **Fit Indices** | **All Children**^a^ | | | | | | | |
| --- | --- | --- | --- | --- | --- | --- | --- | --- |
|  | **2/3 years**^b^ | **4/5 years** | **6/7 years** | **8/9 years** | **10/11 years** | **12/13 years** | **14/15 years** | **16/17 years** |
| **Model A** |  |  |  |  |  |  |  |  |
| RMSEA | 0.075 | 0.096 | 0.104 | 0.102 | 0.109 | 0.099 | 0.113 | 0.106 |
| RMSEA 90% CI | 0.072-0.077 | 0.094-0.097 | 0.103-0.105 | 0.100-0.103 | 0.108-0.111 | 0.097-0.100 | 0.111-0.115 | 0.104-0.108 |
| CFI | 0.912 | 0.884 | 0.898 | 0.887 | 0.895 | 0.905 | 0.917 | 0.903 |
| TLI | 0.898 | 0.866 | 0.885 | 0.873 | 0.882 | 0.893 | 0.906 | 0.891 |
| **Model B** |  |  |  |  |  |  |  |  |
| RMSEA | – | 0.071 | 0.092 | 0.091 | 0.102 | 0.090 | 0.107 | 0.100 |
| RMSEA 90% CI | – | 0.070-0.073 | 0.091-0.093 | 0.089-0.092 | 0.101-0.104 | 0.088-0.091 | 0.105-0.109 | 0.098-0.102 |
| CFI | – | 0.923 | 0.922 | 0.912 | 0.910 | 0.923 | 0.927 | 0.916 |
| TLI | – | 0.911 | 0.910 | 0.898 | 0.897 | 0.911 | 0.917 | 0.903 |
| **Model C** |  |  |  |  |  |  |  |  |
| RMSEA | – | 0.097 | 0.099 | 0.098 | 0.102 | 0.091 | 0.103 | 0.091 |
| RMSEA 90% CI | – | 0.095-0.098 | 0.098-0.101 | 0.096-0.099 | 0.101-0.104 | 0.091-0.093 | 0.101-0.105 | 0.089-0.093 |
| CFI | – | 0.899 | 0.922 | 0.909 | 0.922 | 0.929 | 0.942 | 0.937 |
| TLI | – | 0.882 | 0.910 | 0.896 | 0.911 | 0.919 | 0.934 | 0.928 |
| **Model D** |  |  |  |  |  |  |  |  |
| RMSEA | 0.064 | 0.062 | 0.082 | 0.083 | 0.091 | 0.079 | 0.093 | 0.081 |
| RMSEA 90% CI | 0.061-0.067 | 0.060-0.064 | 0.080-0.083 | 0.081-0.084 | 0.090-0.093 | 0.077-0.080 | 0.091-0.095 | 0.078-0.083 |
| CFI | 0.943 | 0.950 | 0.948 | 0.937 | 0.939 | 0.948 | 0.954 | 0.952 |
| TLI | 0.932 | 0.940 | 0.939 | 0.926 | 0.929 | 0.940 | 0.946 | 0.943 |

Abbreviations: RMSEA, root mean square error of approximation; CFI, comparative fit index; TLI, Tucker-Lewis index; CI, confidence interval.

^a^ All children included children with special healthcare needs and typically functioning children.

^b^ For children aged 2-3 years, the measurement models for Models A and B and for Models C and D were the same because only 1 School Functioning item was included in the LSAC dataset.

**Table a2. Factor Loadings for Model D Across Age Groups for All Children**

| **Item** | **All Children**^a^ | | | | | | | |
| --- | --- | --- | --- | --- | --- | --- | --- | --- |
|  | **2/3 years** | **4/5 years** | **6/7 years** | **8/9 years** | **10/11 years** | **12/13 years** | **14/15 years** | **16/17 years** |
| Phys 1 | 0.901 | 0.900 | 0.907 | 0.885 | 0.924 | 0.897 | 0.922 | 0.899 |
| Phys 2 | 0.884 | 0.945 | 0.968 | 0.922 | 0.928 | 0.909 | 0.915 | 0.891 |
| Phys 3 | 0.777 | 0.818 | 0.933 | 0.917 | 0.943 | 0.927 | 0.947 | 0.910 |
| Phys 4 | 0.576 | 0.598 | 0.715 | 0.699 | 0.765 | 0.792 | 0.851 | 0.849 |
| Phys 5 | 0.585 | 0.674 | 0.757 | 0.742 | 0.876 | 0.829 | 0.929 | 0.860 |
| Phys 6 | 0.547 | 0.481 | 0.629 | 0.657 | 0.668 | 0.653 | 0.771 | 0.622 |
| Pain^b^ | – | – | – | – | – | – | – | – |
| Fatigue^b^ | – | – | – | – | – | – | – | – |
| Emot 1 | 0.662 | 0.714 | 0.739 | 0.759 | 0.791 | 0.785 | 0.833 | 0.839 |
| Emot 2 | 0.712 | 0.731 | 0.758 | 0.792 | 0.802 | 0.836 | 0.861 | 0.856 |
| Emot 3 | 0.641 | 0.621 | 0.654 | 0.678 | 0.698 | 0.714 | 0.779 | 0.728 |
| Emot 4 | 0.487 | 0.507 | 0.590 | 0.621 | 0.614 | 0.639 | 0.707 | 0.702 |
| Emot 5 | 0.789 | 0.778 | 0.708 | 0.747 | 0.769 | 0.793 | 0.814 | 0.817 |
| Soc 1 | 0.720 | 0.732 | 0.768 | 0.763 | 0.781 | 0.784 | 0.803 | 0.799 |
| Soc 2 | 0.772 | 0.775 | 0.752 | 0.822 | 0.829 | 0.847 | 0.852 | 0.855 |
| Soc 3 | 0.723 | 0.728 | 0.724 | 0.792 | 0.795 | 0.804 | 0.845 | 0.810 |
| Soc 4 | 0.777 | 0.753 | 0.727 | 0.775 | 0.751 | 0.799 | 0.819 | 0.822 |
| Soc 5 | 0.775 | 0.779 | 0.886 | 0.833 | 0.872 | 0.847 | 0.862 | 0.877 |
| School 1 | N/A^c^ | N/A^c^ | 0.845 | 0.862 | 0.852 | 0.883 | 0.909 | 0.882 |
| School 2 | N/A^c^ | N/A^c^ | 0.666 | 0.718 | 0.684 | 0.773 | 0.754 | 0.761 |
| School 3 | –^d^ | –^d^ | 0.959 | 0.904 | 0.934 | 0.874 | 0.891 | 0.859 |
| SchAbs 1 | N/A^e^ | 0.864 | 0.783 | 0.802 | 0.807 | 0.790 | 0.845 | 0.880 |
| SchAbs 2 | N/A^e^ | 0.874 | 0.925 | 0.890 | 0.892 | 0.876 | 0.853 | 0.823 |

Abbreviations: Phys, Physical Functioning; Emot, Emotional Functioning; Soc, Social Functioning; School, School Functioning; SchAbs, School Absence; N/A, not applicable.

^a^ All children included children with special healthcare needs and typically functioning children.

^b^ Pain and Fatigue are single item dimensions so could not be empirically tested using confirmatory factor analysis.

^c^ School 1 and School 2 are not included in the parent proxy-report version of the PedsQL for young children.

^d^ School 3 was a single item dimension for children aged 2-5 years since School 1 and School 2 are not included in the PedsQL for those age groups, thus the School Functioning dimension could not be empirically tested using confirmatory factor analysis for age groups 2-3 years and 4-5 years.

^e^ SchAbs 1 and SchAbs 2 were not administered for children aged 2-3 years in the LSAC.
